# Comprehensive Structural and Substrate Specificity Classification of the *Saccharomyces cerevisiae* Methyltransferome

**DOI:** 10.1371/journal.pone.0023168

**Published:** 2011-08-09

**Authors:** Tomasz Wlodarski, Jan Kutner, Joanna Towpik, Lukasz Knizewski, Leszek Rychlewski, Andrzej Kudlicki, Maga Rowicka, Andrzej Dziembowski, Krzysztof Ginalski

**Affiliations:** 1 Laboratory of Bioinformatics and Systems Biology, Interdisciplinary Centre for Mathematical and Computational Modelling, University of Warsaw, Warsaw, Poland; 2 BioInfoBank Institute, Poznan, Poland; 3 Department of Biochemistry and Molecular Biology, University of Texas Medical Branch, Galveston, Texas, United States of America; 4 Institute for Translational Sciences, University of Texas Medical Branch, Galveston, Texas, United States of America; 5 Institute of Biochemistry and Biophysics, Polish Academy of Sciences, Warsaw, Poland; The Centre for Research and Technology, Hellas, Greece

## Abstract

Methylation is one of the most common chemical modifications of biologically active molecules and it occurs in all life forms. Its functional role is very diverse and involves many essential cellular processes, such as signal transduction, transcriptional control, biosynthesis, and metabolism. Here, we provide further insight into the enzymatic methylation in *S. cerevisiae* by conducting a comprehensive structural and functional survey of all the methyltransferases encoded in its genome. Using distant homology detection and fold recognition, we found that the *S. cerevisiae* methyltransferome comprises 86 MTases (53 well-known and 33 putative with unknown substrate specificity). Structural classification of their catalytic domains shows that these enzymes may adopt nine different folds, the most common being the Rossmann-like. We also analyzed the domain architecture of these proteins and identified several new domain contexts. Interestingly, we found that the majority of MTase genes are periodically expressed during yeast metabolic cycle. This finding, together with calculated isoelectric point, fold assignment and cellular localization, was used to develop a novel approach for predicting substrate specificity. Using this approach, we predicted the general substrates for 24 of 33 putative MTases and confirmed these predictions experimentally in both cases tested. Finally, we show that, in *S. cerevisiae*, methylation is carried out by 34 RNA MTases, 32 protein MTases, eight small molecule MTases, three lipid MTases, and nine MTases with still unknown substrate specificity.

## Introduction

Methyltransferases (MTases) comprise a highly important class of enzymes that is present in all living organisms. MTases are involved in various cellular processes, including chromatin remodeling, DNA repair, development and signalling [1], [2]. They act by catalyzing the transfer of a methyl group from mainly S-Adenosyl-L-methionine (known as AdoMet or SAM), to a nucleophilic acceptor, usually a nitrogen or oxygen atom within proteins, nucleic acids, small molecules and lipids.

Protein methylation is the second most common posttranslational modification after phosphorylation, mainly affecting the ε-amine group of lysine and the ω or δ guanidine nitrogen of arginine [3]. The primary targets of protein methylation in *S. cerevisiae* are histones, cytochrome C, ribosomal proteins and various translation factors. Addition of a methyl group to an amino acid residue shields the negative charge, increases the hydrophobicity and introduces steric clashes. Methylation changes both protein-protein and protein-nucleic acid interactions, influencing protein localization, ribosome assembly, RNA processing, protein translation, protein metabolism and cell signalling [4]. Methylation of N-terminal histone tails plays an important role in transcriptional activation or repression, and is directly involved in differentiation, imprinting and X chromosome inactivation [5], [6].

In *S. cerevisiae*, only RNA (not DNA) is methylated enzymatically, both base (mN) and ribose (2′-O-methylation, Nm) groups. Various RNA methylation sites in *S. cerevisiae* have been identified, including those present in rRNA (55 Nm and 10 mN), tRNA (6 Nm, 17 mN and 1 yW (wybutosine)), mRNA (2 mN), sno/snRNA, and telomerase RNA (1 mN) [7]. Importantly, rRNA ribose methylation is undertaken by MTases complexed with snoRNA (snoRNPs) [8]. Despite the fact that all of the guide snoRNPs, and most of the remaining rRNA MTases, in yeast have already been described [9], little is known about the exact role played by rRNA methylation. The functions of induced, single structural changes in rRNA are unclear; however, when they are combined, they are crucial for ribosomal processing and maturation [10]. Methylation of tRNA is essential for proper folding, stabilization and codon recognition [11], whereas cap methylation (m^7^G) is an essential step in mRNA synthesis [12]. The function of additional m^6^A methylations within mRNA is not clear [13]. Telomerase RNA and sno/snRNA are hypermethylated at the cap; however, the biological significance of this modification is also unknown [14], [15].

In *S. cerevisiae*, small molecules and lipids are usually methylated within metabolic pathways, such as ergosterol [16], pyrimidine deoxyribonucleotides [17], phospholipid [18] and siroheme biosynthesis [19]. Phospholipid methylation is critical for the fluidity of cell membranes, which enables receptor mobility. The modification of lipids and small molecules mainly involves C-methylation, although N- and O-methylation have also been reported.

Various experimental and theoretical studies have identified several dozen MTases in *S. cerevisiae*, the majority of which are AdoMet-dependent. A recent bioinformatic study classified AdoMet-dependent MTases into five main structural groups according to their adopted fold, the most common being the Rossmann-like [20]. Interestingly, the genes coding MTases with Rossmann-like fold make up 0.6%–1.6% of the whole genomes [21]. The remaining groups include methionine synthase fold MTases [22], tetrapyrrole methylases [23], SPOUT MTases [24] and SET domain MTases [25]. Although *S. cerevisiae* is a well-studied eukaryotic model organism, we still lack comprehensive knowledge of the MTases encoded by its genome (methyltransferome). For instance, there are a number of known methylation sites within *S. cerevisiae* proteins (e.g., eEF1A), 25S rRNA (7 sites) and tRNAs (2 sites), that have no associated MTase. Moreover, many potential yeast MTases still lack an experimental verification and substrate specificity assignment. In this study, we present a detailed picture of the *S. cerevisiae* methyltransferome, including its comprehensive structural and substrate specificity classification. We also identify previously unknown members of this class of enzymes and present a novel approach to predicting MTase substrate specificity based mainly on the Yeast Metabolic Cycle (YMC) gene expression data [26]. Finally, we show for the first time the correlation between substrate specificity of *S. cerevisiae* MTases and their time within the YMC.

## Results and Discussion

### Identification of the *S. cerevisiae* methyltransferome

We started with an initial set of known MTase families and structures and performed exhaustive transitive Meta-BASIC [27] searches against various protein databases, including the whole *S. cerevisiae* proteome. Meta-BASIC is a highly sensitive method for remote homology detection, capable of finding distant similarities between related proteins that are usually undetectable with standard bioinformatic tools such as PSI-BLAST [28] or RPS-BLAST [29]. This approach enabled us to identify the *S. cerevisiae* methyltransferome (Table 1), which consists of 86 proteins. Fifty three of these proteins were already described in the literature as MTases with biochemically verified MTase activity. Another 32 proteins are putative MTases that were identified in previous bioinformatic studies [21], [30], [31], or were automatically assigned by servers connected to Saccharomyces Genome Database (SGD) [32]. Although many of these proteins are already listed in yeast databases as putative MTases, their enzymatic activity has not been confirmed. We also identified one completely novel MTase (YIL096C) among the hypothetical proteins with unknown function.

**Table 1:** The *S. cerevisiae* methyltransferome.

Fold	Substrate		MTase
Rossmann-like	Protein		YBR034C (HMT1), YBR133C (HSL7), YDR140W (MTQ2), YDR435C (PPM1), YDR440W (DOT1), YDR465C (RMT2), YNL063W (MTQ1), *YBR261C (TAE1)*, *YBR271W*, *YDR316W (OMS1)*, *YIL064W (SEE1)*, *YIL110W (HPM1)*, *YJR129C*, *YKL155C (RSM22)*, *YLR285W (NNT1)*, *YNL024C*, *YOR239W (ABP140)*
	RNA	tRNA	YBL024W (TRM4), YDL201W (TRM8), YBR061C (TRM7), YDR120C (TRM1), YHR070W (TRM5), YJL125C (GCD14), YKR056W (TRM2), YML014W (TRM9), YOL124C (TRM11), YOL125W (TRM13), YOL141W (PPM2), YPL030W (TRM44)
	rRNA		YCL054W (SPB1), YCR047C (BUD23), YDL014W (NOP1), YGL136C (MRM2), YPL266W (DIM1)
	tRNA/rRNA		*YDR083W (RRP8)*, *YNL022C (RCM1)*, *YNL061W (NOP2)*, *YBR141C*, ***YIL096C***, *YLR063W*
	mRNA		YBR236C (ABD1), YGL192W (IME4)
	telomerase/sn/snoRNA		YPL157W (TGS1)
	Small molecule		YER175C (TMT1), YML110C (COQ5), YOL096C (COQ3)
	Lipid		YML008C (ERG6)
	Unknown		*YCL055W (KAR4)*, *YGR001C (AML1)*, *YHR209W (CRG1)*, *YMR209C*, *YMR228W (MTF1)*, *YNL092W*, *YBR225W*, *YKL162C*, *YLR137W*
SPOUT	RNA	tRNA	YDL112W (TRM3), YOL093W (TRM10)
	rRNA		YLR186W (EMG1), YOR201C (MRM1)
	tRNA/rRNA		*YGR283C, YMR310C, YOR021C*
SET domain	Protein		YBR030W (RKM3), YDR198C (RKM2), YDR257C (RKM4), YHR109W (CTM1), YHR119W (SET1), YJL168C (SET2), YPL208W (RKM1), *YHR207C (SET5), YPL165C (SET6), YHL039W (EFM1), YJL105W (SET4), YKR029C (SET3)*
SSo0622-like	RNA	tRNA	YGL050W (TYW3)
Transmembrane	Protein		YDR410C (STE14)
	Lipid		YGR157W (CHO2), YJR073C (OPI3)
Tetrapyrrole methylase	Protein		YLR172C (DPH5)
	Small molecule		YKR069W (MET1)
DNA/RNA-binding 3-helical bundle	Protein		YDL200C a (MGT1)
TIM beta/alpha-barrel	Small molecule		YER091C^b^ (MET6), YLL062C (MHT1), YPL273W (SAM4)
Thymidylate synthetase	Small molecule		YOR074C^c^ (CDC21)

MTases are grouped according to the structural similarity of their catalytic domains (Fold) and substrate specificity (Substrate). Known MTases with experimentally determined substrate specificity are shown in regular font, putative MTases are italicized, and the newly detected MTase is highlighted in bold. Non-periodic MTases are underlined. With the exception of ^a^DNA, containing 6-O-methylguanine, ^b^5-methyltetrahydropteroyltri-L-glutamate, and ^c^5,10-methylenetetrahydrofolate, AdoMet is the methyl group donor.

### Structural classification

**Figure 1:**
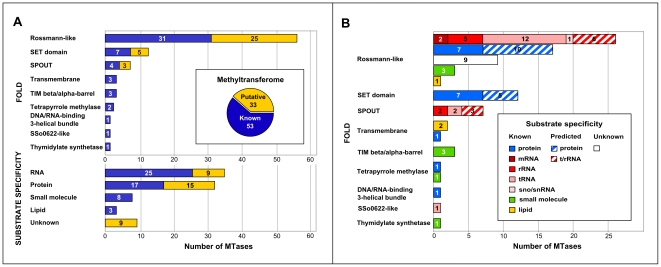
Comprehensive picture of the *S. cerevisiae* methyltransferome. (A) Structural (fold) and substrate specificity classifications. Eighty-six MTases (known MTases with experimentally verified activity and putative MTases identified in previous bioinformatic studies, including one newly detected here) were divided into several groups based on similarity of their structure (within catalytic domain) and substrate. (B) Detailed structural vs. substrate specificity classification.

Using 3D-Jury method of consensus fold recognition [33], we confidently predicted the 3D structure of the catalytic domains in all of the identified *S. cerevisiae* MTases, for which the structure had not been solved experimentally (83 MTases in total). As a result, we structurally classified all 86 MTases and found that their catalytic domains adopt up to 9 different folds (Figure 1A), significantly more than previously reported [20]. The most common scaffold for methyl transfer is the Rossmann-like fold, found in 56 MTases. This is many more than described by Petrossian and Clarke [34], who used HMM profiles and combinatorial motif scanning for Rossmann-like MTases to identify 32 known and potentially several putative MTases in the yeast proteome.

Less commonly observed folds for methyl transfer in *S. cerevisiae* include SET domain (12 MTases), SPOUT (7 MTases), TIM beta/alpha-barrel (3 MTases), transmembrane (3 MTases), tetrapyrrole methylase (2 MTases), DNA/RNA-binding 3-helical bundle (1 MTase), SSo0622-like (1 MTase), and thymidylate synthetase (1 MTase). Consequently, the majority of *S. cerevisiae* MTases are alpha/beta proteins: Rossmann-like, SPOUT, TIM beta/alpha-barrel and tetrapyrrole methylase. However, other architectures were also found, such as alpha+beta (SSo0622-like and thymidylate synthase), all beta (SET domain), all alpha (DNA/RNA-binding 3-helical bundle) and transmembrane proteins.

Our results suggest that methylation in *S. cerevisiae* almost exclusively uses AdoMet as a methyl group donor; only three MTases use other compounds (Table 1). The protein MTase, MGT1 (DNA/RNA-binding 3-helical bundle fold), uses DNA containing 6-O-methylguanine as a methyl group donor, while two small molecule MTases, MET6 (TIM beta/alpha-barrel fold), and CDC21 (thymidylate synthetase fold) use 5-methyltetrahydropteroyltri-L-glutamate and 5,10-methylenetetrahydrofolate, respectively. Interestingly, the different AdoMet-dependent MTase folds do not show any common features within the local architecture around the AdoMet binding site. In addition, AdoMet can be bound in significantly different conformations such as “extended” (Rossmann-like) or “bend” (SPOUT, SET domain and tetrapyrrole methylase).

**Figure 2:**
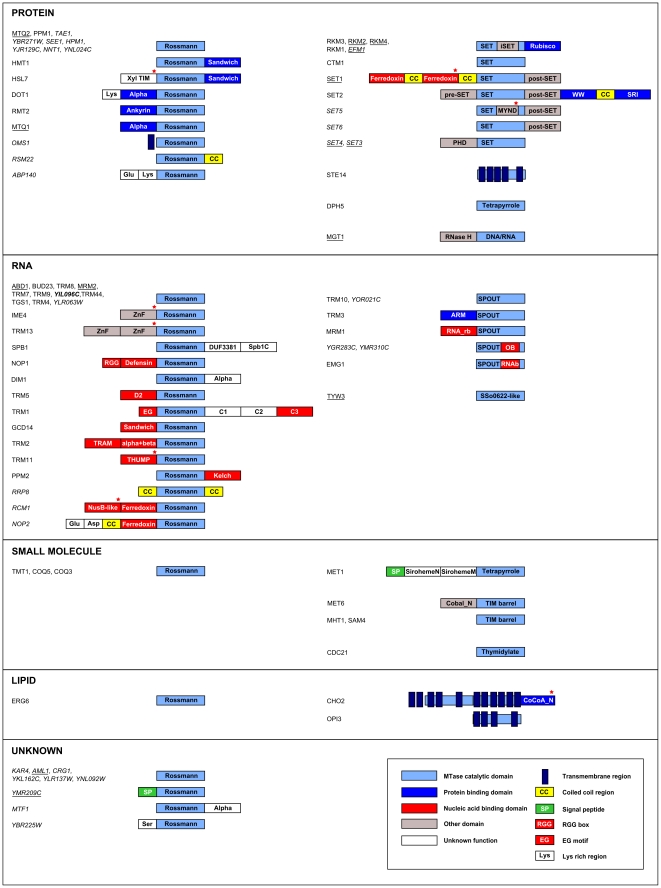
Domain architecture of *S. cerevisiae* MTases. The MTases were grouped according to their common substrate specificities (e.g., protein, RNA, small molecule or lipid) and the fold of catalytic domain. Known MTases with experimentally determined substrate specificity are shown in a regular font, putative MTases in italics, and newly detected MTase in bold. Non-periodic MTases are underlined. The new domains identified in this study are marked with a red asterisk. Sandwich, beta sandwich; Xyl TIM, TIM beta/alpha-barrel belonging to the Xylose isomerase-like superfamily; Alpha, α-helical domain; Ankyrin, Ankyrin repeats; ZnF, zinc finger; Spb1C, Spb1 C-terminal domain; Defensin, defensin-like fold; iSET, SET-inserted domain; Rubisco, Rubisco LSMT C-terminal-like domain; SRI, SET2 Rpb1 interacting domain; PHD, PHD zinc finger; DNA/RNA, DNA/RNA-binding 3-helical bundle; RNase H, RNase H-like domain; ARM, ARM repeat; RNA_rb, RNA ribose binding domain; SirohemeN, Siroheme synthase N-terminal domain-like; SirohemeM, Siroheme synthase middle domain-like; Cobal_N, Cobalamin-independent synthase N-terminal domain; CoCoA_N, Calcium binding and coiled-coil domain-like (N-terminal); OB, OB-fold domain; RNAb, RNA binding domain.

At least half of the *S. cerevisiae* MTases possess additional domains that are mainly used for protein or RNA binding (Figure 2). Using distant homology detection and fold recognition, we structurally and functionally annotated the additional domains and identified several new domain architectures. The two most interesting predictions are described below.

#### SET5 (YPL165C)

The Smyd protein family comprises genes found in Metazoa, Fungi and plants, but has not yet been described in S. cerevisiae. Smyd proteins are involved in the transcriptional regulation of cellular proliferation and differentiation, as well as in cancer development [35], [36]. We found that SET5 contains an MYND (MYeloid, Nervy and DEAF-1) domain (Figure 2) composed of two zinc fingers that are involved in the recruitment of histone deacetylase-containing complexes. This domain, together with the SET and post-SET domains that are also present in SET5, is characteristic feature of Smyd proteins. This suggests that the S. cerevisiae SET5 MTase, similarly to other Smyds, may be involved in histone methylation (gene silencing coupled with histone deacetylase activity).

#### CHO2 (YGR157W)

Surprisingly, we identified the N-terminal-like domain of the coiled-coil coactivator (CoCoA) within the C-terminus of the transmembrane MTase, CHO2 (Figure 2). This domain mediates transcriptional activation by the β-catenin coactivator in the Wnt signalling pathway [37]. CHO2 is localized to the endoplasmic reticulum (ER), with the N-terminal-like CoCoA domain predicted to be positioned inside the ER lumen. In addition, CHO2 contains a highly conserved sequence motif (DWIGLYKV) that, in CoCoA, interacts with p300, a cofactor that participates in transcriptional activation [37]. Although the Wnt signalling pathway is not present in yeast, detection of an N-terminal-like CoCoA domain in the CHO2 MTase suggests the existence of a previously unknown regulation mechanism that may be involved in chromatin-mediated reshaping of the ER during nuclear membrane formation [38].

### Substrate specificity classification

**Figure 3:**
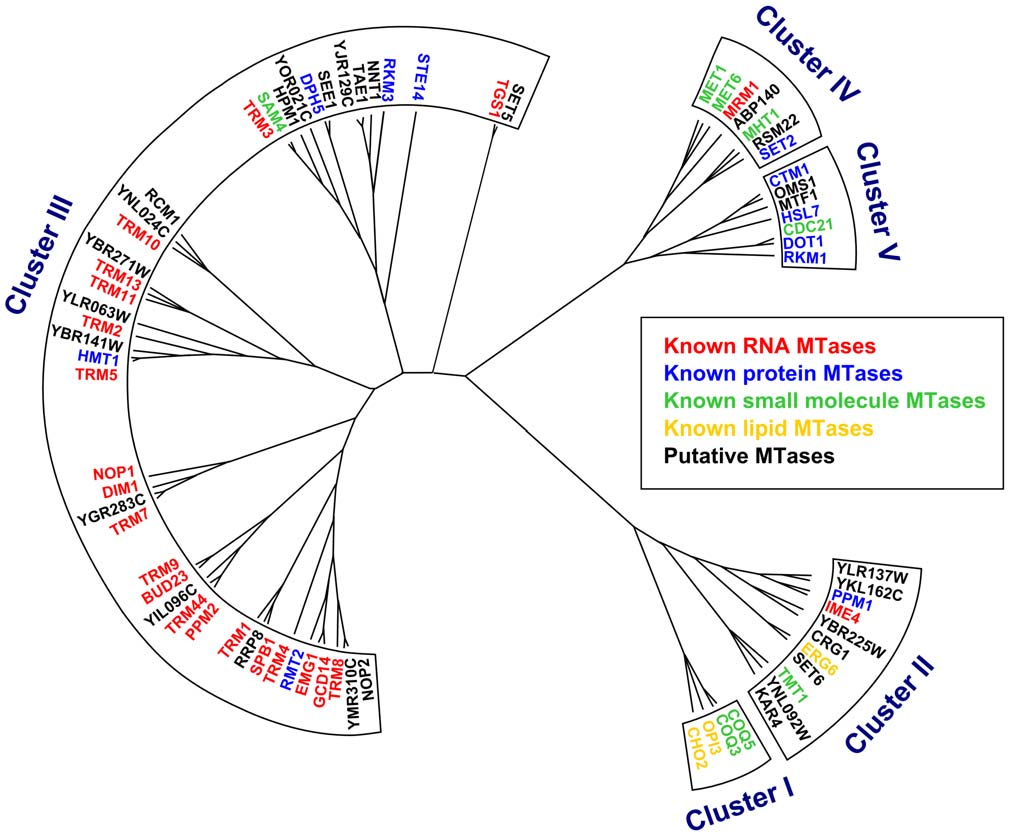
Hierarchical clustering tree for all *S. cerevisiae* periodic MTases. Seventy-two periodic MTases were divided into five clusters, each containing MTases with similar expression profiles during the Yeast Metabolic Cycle (YMC). Branch lengths correspond to correlation coefficients of gene expression profiles during the YMC obtained from SCEPTRANS.

**Figure 4:**
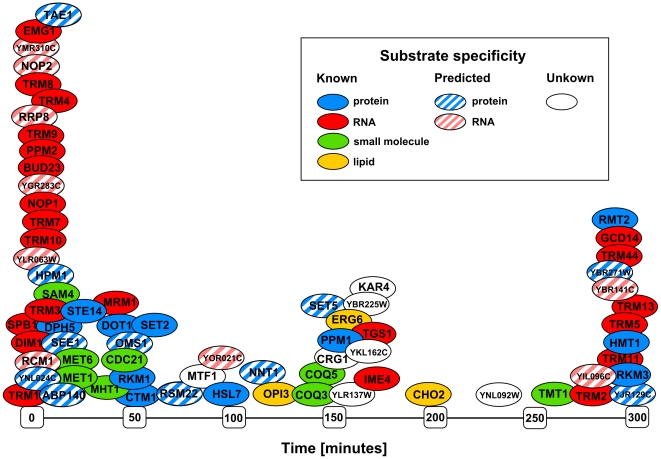
Metabolic cycle-dependent expression of *S. cerevisiae* periodic MTases. Each MTase is positioned at its gene expression peak within the YMC (which lasts 300 min).

We also classified *S. cerevisiae* MTases according to their substrate specificity by dividing them into four general groups: protein MTases, RNA MTases, lipid MTases and small molecule MTases. For the 53 MTases with known substrate specificity, these groups comprise 17, 25, three and eight proteins, respectively (Figure 1B). To predict the substrate specificity of the 33 putative *S. cerevisiae* MTases embracing 25 Rossmann-like, five SET domain and three SPOUT fold proteins, we developed a novel theoretical approach. The method exploits the characteristic gene expression patterns observed for the different classes of MTases in the YMC. The list of 51 MTases, reported as periodic by Tu *et al.* [26], was extended based on a visual examination of temporal expression profiles at SCEPTRANS web server [39]. This procedure yielded 72 MTases (44 with known substrate specificity and 28 putative MTases), to which we are referring as “periodic MTases” in this paper (Table 1). We used the expression profiles seen during the YMC, since this system is known for “compartmentalization in time” [26]. This means that genes with similar functions tend to be expressed within a specific temporal window during the YMC. Indeed, hierarchical clustering of MTases based on the correlation between their gene expression profiles during the YMC, results in clusters enriched in MTases with certain substrate specificities (Figure 3). Particularly, cluster III comprises mainly of tRNA MTases, rRNA MTases, and a few protein MTases that methylate ribosomal proteins. The statistically significant grouping of tRNA and rRNA MTases within this cluster is supported by enrichment p-value (7×10^−6^) calculated from hypergeometric distribution. We also found that these periodic MTases are expressed in a very short time window (35 min) during the YMC (300 min), what is not seen for any other yeast MTases (Figure 4).

RNA MTases have statistically very significant tendency to have high maximum isoelectric point (max pI) (see Materials and Methods): 96% RNA MTases have max (i.e. local) pI higher than 8 (p-value 2×10^−7^, hypergeometric). This is not unexpected, as protein domains involved in binding nucleic acids are characterized by the presence of extensive number of positively charged residues, resulting in their high pI. On the contrary, protein MTases exhibit statistically significant preference for low max pI: 65% protein MTases have max pI lower than 8 (p-value 0.01, hypergeometric). Therefore, the calculated max pI values can aid in distinguishing between RNA and protein MTases. Cellular localization is also informative, for the same reason that it is being used to filter protein-protein interaction data: it can help determine if two molecules potentially able to interact have a chance to be in proximity (the same organelle) in vivo. For example, all three lipid MTases are localized in the ER (p-value 2×10^−4^, hypergeometric). Fold is bringing even more information about MTase substrate specificity and in some cases it is the only information needed. While Rossmann-like fold MTases can methylate all types of substrates (proteins, RNA, small molecules and lipids), SPOUT [24] and SET domain [25] MTases seem to methylate only RNA and proteins, respectively.

We combined all described above relationships between the YMC expression profiles, max pI, subcellular localization, fold assignment and the type of MTase substrate, to propose a novel approach for predicting substrate specificity. This heuristic method was implemented using decision tree (Table 2), validated on 44 known periodic MTases. We applied this approach to 28 putative periodic MTases, and predicted the substrate specificity for 19 (16 Rossmann-like, two SPOUT and one SET domain) of them, leading to the prediction of 11 novel protein MTases (YHR207C, YBR271W, YJR129C, YNL024C, YLR285W, YIL064W, YIL110W, YOR239W, YKL155C, YDR316W and YBR261C) and eight novel RNA (tRNA or rRNA) MTases (YLR063W, YBR141C, YIL096C, YNL022C, YNL061W, YDR083W, YMR310C and YGR283C). Our prediction regarding the RNA substrate specificity for YIL096C (a newly identified MTase in this study) is consistent with experimental data indicating that this protein associates with 60S ribosomal subunit precursors, and is potentially involved in ribosome biogenesis [40], [41]. Taken together, these results suggest that YIL096C is a novel rRNA MTase.

**Table 2:** Decision tree rules used for predicting MTase substrate specificity.

Substrate	Max pI	Cluster	Fold	Localization	R	P	F	N
Protein	<8	III or V	Rossmann-like, SET domain, Tetrapyrrole methylase or Transmembrane	not mitochondrial	91%	100%	0.95	11
	≥8	IV or V	Rossmann-like or SET domain	not ER				
tRNA or rRNA	≥8	III			90%	95%	0.92	8
Small molecule	<8	I or IV			62%	100%	0.77	0

Decision tree rules were based on the maximum isoelectric point (Max pI), similarity of expression profiles (Cluster; the numbering of clusters that group MTases with similar gene expression profiles is given in Figure 3), fold assignment (Fold) and cellular localization (Localization). Columns R, P and F show the Recall, Precision and F-measure values calculated for each substrate specificity class with non-zero R. The number of MTases with the substrate specificity predicted by each of the rules is shown in column N.

For the five non-periodic and nine periodic MTases lacking predicted substrate based on decision tree, we based our predictions on fold assignment only. Consequently, using fold assignment for the catalytic domains, we predicted the substrate specificity for all the remaining putative SPOUT and SET domain fold MTases, including three non-periodic (YHL039W, YJL105W and YKR029C, predicted to be protein MTases) and two periodic (YPL165C and YOR021C, predicted to be protein and RNA MTases, respectively).

Altogether, we predicted the substrate specificity of 24/33 putative MTases, and identified 15 new protein MTases and nine new RNA MTases; thus, significantly increasing our knowledge regarding methylation in yeast (Table 1). Finally, our classification shows that the *S. cerevisiae* methyltransferome embraces 32 protein MTases, 34 RNA MTases, eight small molecule MTases, three lipid MTases and nine Rossmann-like fold MTases with, as yet, unknown substrate specificity (Figure 1B).

### Experimental verification

**Figure 5:**
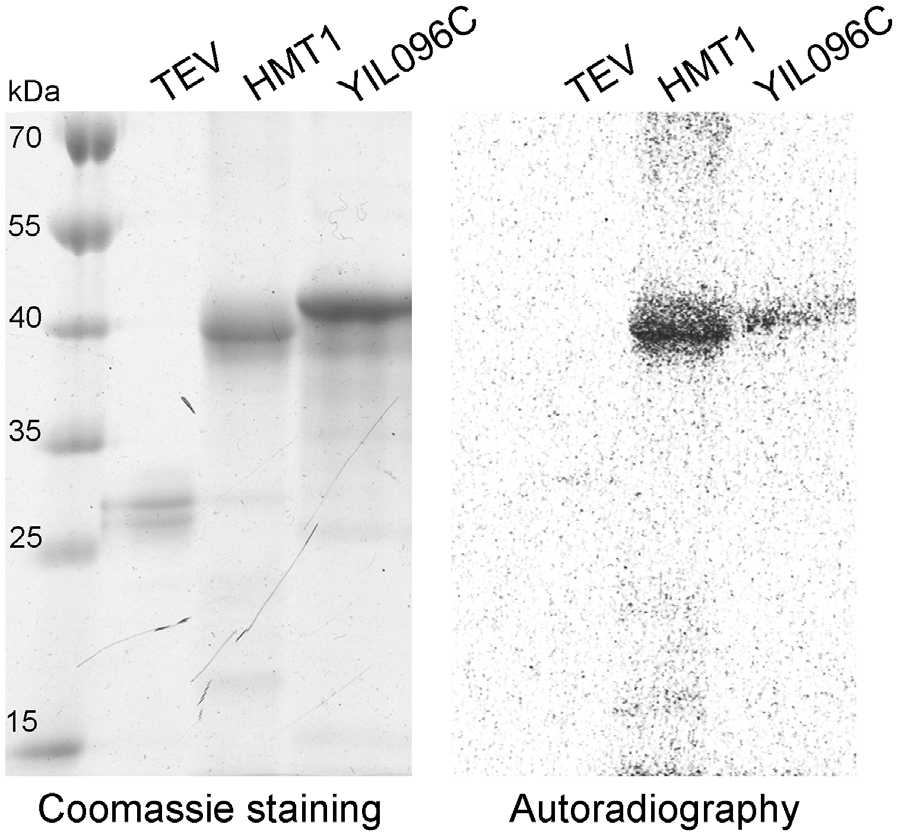
Putative MTase YIL096C binds AdoMet. Purified YIL096C (with HIStagSUMO), HTM1 and TEV protease were exposed to UV light in the presence of [^3^H] AdoMet. Both Coomassie stained proteins (left panel) and the autoradiography of crosslink products (right panel) are shown. HMT1 (known MTase) and TEV protease were used as positive and negative controls, respectively.

Firstly, we analyzed whether YIL096C, identified here as novel MTase, is able to bind a methyl group donor, using UV crosslinking [31], [42]. Recombinant YIL096C protein was thus exposed to UV light in the presence of [^3^H] AdoMet. The crosslink product was detected on a tritium screen (Figure 5) providing that AdoMet can bind both to YIL096C and HMT1 (known MTase used as positive control) but not TEV protease (negative control).

**Figure 6:**
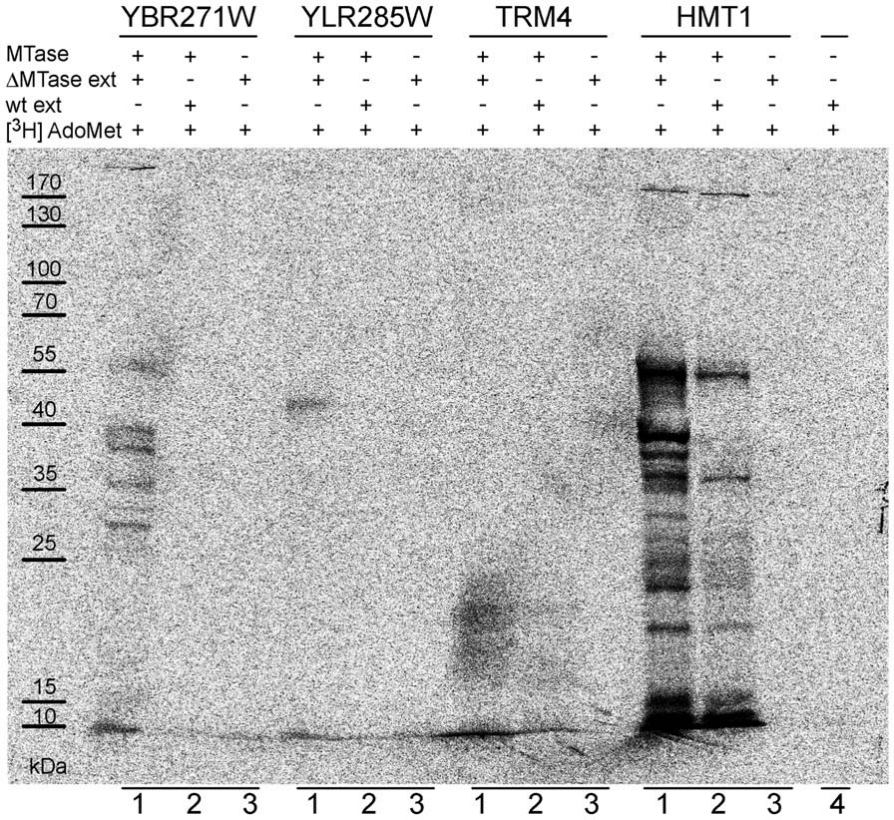
YBR271W and YLR285W (NNT1) are protein MTases. Recombinant proteins (MTases) were incubated with native yeast extracts from the respective knockout strains (ΔMTase ext) and [^3^H] AdoMet (lane 1). Reaction products were resolved on SDS-PAGE gel and exposed to tritium screen. To test the specificity of these reactions, analyzed proteins were also incubated with yeast extract from the wild-type strain (wt ext) and [^3^H] AdoMet (lane 2). As a control, yeast extracts from knockout and wild-type strains were incubated with [^3^H] AdoMet only (lanes 3 and 4). HMT1 (a protein MTase) and TRM4 (an RNA MTase) were used as positive and negative controls, respectively.

To validate the approach for substrate specificity prediction, we performed MTase activity assays for two *S. cerevisiae* proteins predicted to be Rossmann-like protein MTases: YBR271W and YLR285W (NNT1). Briefly, purified recombinant proteins were incubated with native total cell extracts from the wild-type and respective knockout strains in the presence of tritium-labeled AdoMet. The reaction products were then analyzed by SDS-PAGE followed by autoradiography (Figure 6). The presence of protein methylation products confirmed that YBR271W and YLR285W are protein MTases. Interestingly, YLR285W was previously assigned as a putative nicotinamide N-methyltransferase based on distant similarity to human NNMT [43]. This suggests that a simple sequence comparison approach cannot correctly predict the substrate specificity of Rossmann-like fold MTases. Our data indicate that YBR271W modifies several proteins, while YLR285W appears to have only one specific protein substrate. In both cases, methylated proteins were detected only when the deletion strains were used (Figure 6, lane 1), which strongly suggests that these modifications are stable. Interestingly, YBR271W is predicted to be a part of rRNA and ribosome biosynthesis (RRB) regulon [40] and is also expected, from a genome-wide in vivo screen (PCA) [44], to interact with RPC34, an RNA polymerase III subunit C34, a key determinant in Pol III recruitment. Methylation of RPC34 (Figure 6, one of the observed bands corresponds to the molecular weight of RPC34, which is 36 kDa) would be in agreement with the predicted role of YBR271W in rRNA biosynthesis, because Pol III is responsible for 5S rRNA synthesis. As expected, we also found protein methylation patterns matching known substrates for HMT1, but not for RNA MTase TRM4 (Figure 6, the smear at the bottom of the gel represents tRNA). These results strongly support the theoretical model used in this study to predict the substrate specificity of putative MTases.

### Conclusion

**Figure 7:**
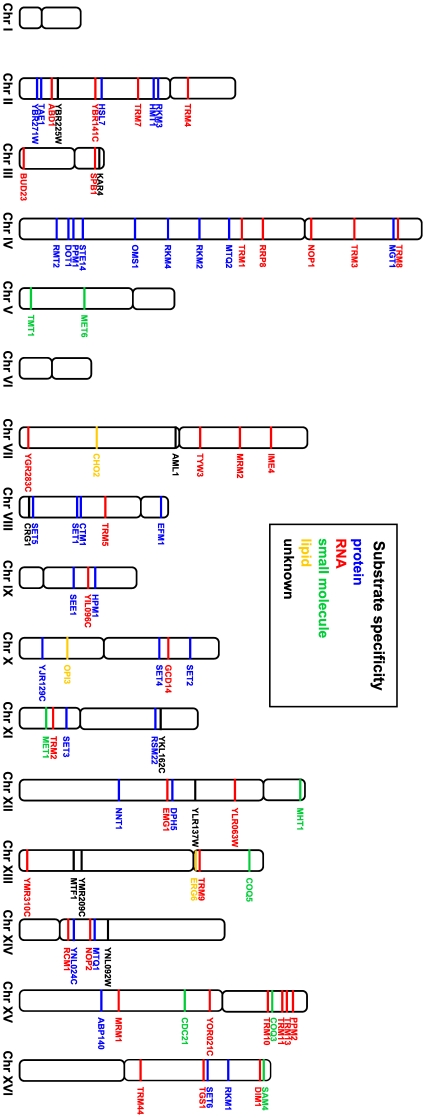
Localization of MTase genes within the *S. cerevisiae* genome. MTases are colored according to their substrate specificity.

Identification of the *S. cerevisiae* methyltransferome, together with its structural and substrate specificity classification, has enabled us to shed new light on enzymatic methylation in yeast. In *S. cerevisiae*, methylation is carried out by 86 MTases among which are 34 RNA MTases, 32 protein MTases, eight small molecule MTases, three lipid MTases and nine MTases with unknown substrate specificity. These MTases may adopt up to nine different folds within the catalytic domain; however, as may be expected, the Rossmann-like fold is the most commonly used scaffold for the methyl transfer reaction. In addition, genes encoding *S. cerevisiae* MTases are almost uniformly distributed across all chromosomes, with the exception of chromosomes I and VI, where they are not present (Figure 7).

It should be noted that prediction of substrate specificity for Rossmann-like fold MTases (25 of 33 putative MTases in *S. cerevisiae*) based on simple sequence similarity is not feasible. Specifically, while SPOUT or SET domain folds seem to have clearly defined substrate specificity (RNA and protein, respectively), Rossmann-like fold MTases perform all types of methylation. For example, PPM1 and PPM2, which share more than 30% sequence identity within their catalytic domains, are protein and tRNA Rossmann-like fold MTases, respectively. We discovered that gene expression profile during the YMC, fold assignment, max pI and protein localization all statistically significantly correlate with the type of MTase substrate and thus can be used to predict MTase substrate specificity. We proposed a simple set of decision rules which allowed to assign general substrates to 24 putative MTases, including 16 of the Rossmann-like fold. We confirmed our predictions experimentally in both cases tested, while in some other cases there is a strong supporting evidence in the literature (e.g. for newly detected RNA MTase, YIL096C). Our results provide the basis for more detailed biochemical analyses of individual MTases and the identification of their specific protein and RNA substrates.

## Materials and Methods

### Identification of the methyltransferome

Initially, both known and putative MTases were selected from various databases, including the Saccharomyces Genome Database (SGD) [32] and the catalogued protein families (PFAM [45], COG and KOG [46]) and structures (PDB [47] and SCOP [48]). The databases were searched using the term “methyl” as a text query and the list of hits was screened manually. The PDB database was also searched using E.C. number 2.1.1., which corresponds to MTase function. The obtained data set was used for further comprehensive searches against the whole *S. cerevisiae* proteome using Meta-BASIC [27], a highly sensitive method for distant homology detection based on the comparison of meta-profiles (sequence profiles enriched with predicted secondary structures). Consequently, novel MTases, which were not present in the initial set, were identified using the Gene Relational DataBase (GRDB) system. GRDB includes pre-calculated Meta-BASIC connections between 11,127 PFAM, 10,361 KOG and COG families, 20,877 proteins of known structure (PDB90; representatives from the PDB database, filtered at 90% sequence identity) and 6719 *S. cerevisiae* proteins (the complete *S. cerevisiae* proteome). Each family, structure and *S. cerevisiae* protein in the system was represented by: (i) its sequence (for *S. cerevisiae* proteins and PDB90) or consensus sequence (for PFAM, COG and KOG families), (ii) its sequence profile generated with PSI-BLAST [28] (3 iterations, inclusion threshold 0.001) using the NCBI non-redundant protein sequence database derivative (NR70), and (iii) its secondary structure, predicted using PSI-PRED [49].

The search strategy was based on the concept of transitivity, where each newly identified PFAM, KOG and COG family, PDB structure, or *S. cerevisiae* protein was used in further Meta-BASIC searches until no new additional MTase hits were found. In addition to the highly reliable Meta-BASIC predictions with scores above 40 (corresponds to E-value <0.05), all hits with scores between 30 and 40 were also considered to identify any potentially correct predictions that may have been placed among the unreliable or incorrect ones. These potentially correct predictions were selected based on the manual assessment of conservation of the core secondary structure elements and sequence motifs deemed critical for the given fold and MTase function.

To confirm any non-trivial predictions that met the above criteria, the corresponding sequences were submitted to the Protein Structure Prediction Meta Server (http://meta.bioinfo.pl), which integrates various top-of-the-line fold recognition methods. The models generated by these methods were analyzed with 3D-Jury [33], a meta-predictor that uses a consensus approach to select the most abundant models. Predictions were deemed reliable when the assigned scores were above a confidence threshold of 50 [50].

### Fold assignment and domain architecture analysis

The *S. cerevisiae* MTases were divided into distinct structural groups based on the structural similarity (fold) of their catalytic domains. The catalytic domain of each MTase was first identified with Meta-BASIC, followed by fold assignment using 3D-Jury and SCOP classification. To detect additional protein domains, sequence fragments (after removal of the catalytic MTase domain) were also analyzed using Meta-BASIC coupled, for non-trivial predictions, with 3D-Jury. Confident assignments were selected based on reliable Meta-BASIC and 3D-Jury scores and conservation of critical secondary structure elements and sequence motifs. Transmembrane regions were predicted using ConPred II [51], Phobius [52] and TOPCONS [53], and only those regions identified by all these methods as “highly probable” were accepted. Coiled coils were detected with Marcoil 1.0 [54], signal peptides with SignalP 3.0 [55] and functionally important motifs with PROSITE [56].

### Prediction of substrate specificity

Information regarding the substrate specificity of the known MTases was obtained from SGD and literature searches. Predictions of general substrate specificity (protein, RNA, lipid and small molecule) of the putative MTases were based on similarities in their gene expression patterns during the YMC, calculated pI, fold assignment and cellular localization. The periodic MTases were selected based on a visual examination of temporal expression profiles at SCEPTRANS [39] (Table 1). Then, a matrix of correlation coefficients of expression profiles for periodic MTases was obtained from the SCEPTRANS and was used to group these MTases into 5 clusters, each consisting of MTases with similar expression profiles during the YMC. The *p*-values of enrichment of MTases with a given substrate specificity was computed using a hypergeometric probability distribution. The results confirmed potential usefulness of this clustering for predicting substrate specificity of periodic MTases. Also, three pI values were calculated for each MTase (for the whole protein, the catalytic domain and the remaining regions) using the ExPASy tool (ProtParam) [57]. The maximum of these three values was defined as max pI. The cellular localization data obtained from SGD was also considered. For MTases with unknown cellular localization, this data was predicted using the consensus of the results obtained from three localization prediction servers (WoLF PSORT [58], BaCelLo [59] and MultiLoc [60]).

The groupings derived from hierarchical clustering for known periodic MTases, together with the maximum pI value, the fold assignment and the cellular localization were used to propose a decision tree. Removing each MTase from the training set (all periodic known MTases) and trying to predict its substrate specificity based on the newly generated decision tree allowed us to assess how dependent the results were upon the training set. Using “leave-one-out” cross-validation allowed for the correct prediction of the general substrate specificity for 75% of the whole training set. The rules used for the prediction of substrate specificity for the periodic putative MTases are listed in Table 2. For each decision rule, the Recall, Precision and F-measures were calculated as follows: Recall = TP/(TP + FN), Precision = TP/(TP + FP), F-measure = 2(Recall*Precision)/(Recall + Precision), where TP, FN and FP represent true-positives, false-negatives and false-positives, respectively. Predicting the substrate specificity of non-periodic putative MTases was based on fold assignment (SPOUT: RNA MTase, SET domain: protein MTase) only.

### Strains and media

The following yeast strains (Euroscarf) were used in this study: BY4741 (*MAT*a *his3*Δ*1 leu2*Δ*0 met15*Δ*0 ura3*Δ*0*), BY4741 ΔYBR271W, BY4741 ΔYLR285W, BY4741 ΔYBR034C (ΔHMT1), and BY4741 ΔYBL024W (ΔTRM4). The standard yeast genetic methods and selective growth media have been previously described [61].

### Protein expression and purification

The putative MTases, YIL096C, YBR271W and YLR285W, along with the HMT1 and TRM4 MTases (controls) were produced in *E. coli* (BL21-CodonPlus-RIL strain) as N-terminal HIStagSUMO tag fusions using LB medium and overnight IPTG inductions at 18°C. The bacterial pellets were lysed by sonication in buffer A (20 mM Tris-HCl pH 8.0, 200 mM NaCl, 10 mM imidazole, 10 mM 2-mercaptoethanol) and purified on His-Trap FF Crude columns (GE Healthcare). The proteins were further purified by size-exclusion chromatography on a Superdex 75 10/300 GL column (GE Healthcare) in buffer containing 10 mM Tris-HCl pH 8.0 and 150 mM NaCl. Finally, glycerol was added to the protein aliquotes (30% final concentration), which were then stored at −80°C. The purity and quantity of the proteins was assessed by SDS-PAGE.

### UV crosslinking

Recombinant proteins (10–25 µg) were mixed with 2 µCi [^3^H] AdoMet (80 Ci/mmol, Hartmann Analytic GmbH) in a buffer containing 10 mM potassium phosphate pH 7.0, 100 mM NaCl, 2 mM EDTA, 1 mM dithiothreitol, 5% glycerol in PCR tube [42]. The reaction mixture was exposed to UV irradiation in a UVC 500 crosslinker (Amersham Bioscience) for 10 min on ice, 3 cm from the light source. The products were run on a 12% SDS-PAGE gel. After Coomassie blue staining, dried gel was exposed to tritium screen (GE Healthcare) for 72 hr at RT.

### *In vitro* methylation assay

Yeast whole-cell extracts were prepared as previously described [62]. Recombinant proteins (5 µg) were incubated with 30 µg of native yeast extract (from a wild-type strain and strain from which the gene encoding the analyzed protein had been deleted) in the presence of [^3^H] AdoMet (0.5 µCi/reaction) in 15 µl of reaction buffer (10 mM HEPES pH 8.0, 2 mM EDTA, 50 mM KCl, 1 mM DTT). Protein extracts were incubated at RT for 1 hr before being diluted 2-fold in Laemmli buffer and resolved on a 12% SDS-PAGE gel. Gel was stained with Coomassie blue, dried and exposed overnight to tritium screen.
